# Targeting Polo-like kinase 1 and TRAIL enhances apoptosis in non-small cell lung cancer

**DOI:** 10.18632/oncotarget.25618

**Published:** 2018-06-19

**Authors:** Alfiah Noor, Ijeoma Adaku Umelo, Peter Kronenberger, Philippe Giron, Elly De Vlieghere, Olivier De Wever, Erik Teugels, Jacques De Grève

**Affiliations:** ^1^ Laboratory of Molecular Oncology and Department of Medical Oncology, Oncologisch Centrum, Universitair Ziekenhuis Brussel, Brussels, Belgium; ^2^ Laboratory of Biotechnology, Department of Healthcare, Erasmushogeschool Brussel, Brussels, Belgium; ^3^ Laboratory of Experimental Cancer Research, Cancer Research Institute Ghent (CRIG), Ghent University, Ghent, Belgium

**Keywords:** NSCLC, TRAIL, PLK1, cell cycle, combination therapy

## Abstract

Tumor necrosis factor-related apoptosis-inducing ligand (TRAIL) can selectively induce apoptosis in cancer cells without causing damage to normal cells. However, some tumors are resistant to TRAIL monotherapy and clinical studies assessing targeted agents towards the TRAIL receptor have failed to show robust therapeutic activity. Evidence has shown that standard anti-mitotic drugs can induce synergistic apoptosis upon combination with TRAIL via cell cycle arrest. Polo like kinase-1 (PLK1) plays a critical role in different stages of cell cycle progression and mitosis. A number of investigations have demonstrated that PLK1 inhibition causes cell cycle arrest and mitotic catastrophe in non-small cell lung cancer (NSCLC), and we thus postulated that PLK1 inhibition could enhance TRAIL-induced apoptosis. We demonstrate that the combination of a TRAIL receptor agonist and a PLK1 inhibitor synergistically reduces cell viability, and strongly increases apoptosis in NSCLC cellular models. Consistent with our *in vitro* observations, this drug combination also significantly reduces tumor growth *in vivo*. Our data additionally reveal that G2/M cell cycle arrest and downregulation of Mcl-1 and signal transducer and activator of transcription 3 (STAT3) activity following PLK1 inhibition may contribute to the sensitization of TRAIL-induced apoptosis in NSCLC. Together, these data support the further exploration of combined TRAIL and PLK1 inhibition in the treatment of NSCLC.

## INTRODUCTION

Lung cancer is the leading cause of cancer-related death worldwide, with 80% of lung cancers classified as non-small cell lung cancer (NSCLC). The identification of somatic mutations in NSCLC has led to the development of targeted therapies. Despite impressive initial results, these treatments are not yet curative, due to the emergence of drug-resistant clones that confer resistance to these targeted agents.

Tumor necrosis factor (TNF) is an apoptotic ligand that was primarily identified as a natural anti-cancer agent, due to its ability to induce apoptosis in cancer cells [1–3]. However, due to its lack of specificity, TNF has demonstrated an unfavorable toxicity profile in patients [4]. Accordingly, the development of agents that more directly target the apoptotic machinery and could lead to better therapeutic efficacy and less toxic side effects has generated a lot of interest. TNF-related apoptosis-inducing ligand (TRAIL) also known as Apo-2 ligand, is a member of the TNF cytokine family. In contrast to TNF, pre-clinical investigations have shown that TRAIL can selectively induce apoptosis in cancer cells without causing damage to normal cells [5–10]. Moreover, TRAIL plays an important role in innate and adaptive immunity and its activity is considered as a natural path to eliminate cancer cells [11]. Therefore, inducing/activating the TRAIL apoptotic pathway is a proposed strategy that has received considerable attention as a potential anti-cancer therapy [12, 13].

Although TRAIL can induce apoptosis in cancer cell lines, clinical trials with several TRAIL receptor-agonists, including recombinant human TRAIL (rhTRAIL), and an agonist monoclonal antibody specific for TRAIL receptors, have failed to show robust therapeutic activity in patients. It has also been shown that most primary cancers are intrinsically TRAIL resistant [14, 15]. In addition, phase II clinical studies have also been disappointing, demonstrating resistance to TRAIL receptor targeted agents in human cancers including NSCLC [16]. A number of studies have reported that targeting TRAIL receptor combined with standard systemic therapy such as cisplatin, camptothecin and paclitaxel, can considerably increase anti-tumor activity in experimental NSCLC models [14]. In addition, anti-mitotic drugs such as doxorubicin or methotrexate induce synergistic apoptosis upon combination with TRAIL via cell cycle arrest [17].

Polo like kinase-1 (PLK1) has received attention as a candidate anti-cancer target as it plays a critical role in different stages of cell cycle progression and mitosis [18] including mitotic entry, G2/M checkpoint, spindle assembly maturation, chromosome segregation, and mitotic exit [19]. PLK1 is overexpressed in several tumor types [20–23] including NSCLC [24, 25]. In contrast, it is almost undetectable in most normal adult tissue [26]. Depleting PLK1 expression by RNA interference has been shown to specifically reduce cellular growth, induce mitotic arrest and strongly induce apoptosis in cancer cells including NSCLC cells, in particular when Ras mutated [25, 27–29]. PLK1 inhibition, furthermore, causes cell cycle arrest and mitotic catastrophe in NSCLC [30, 31].

We thus hypothesized that the combined targeting of two essential pathways for the malignant phenotype, namely the cell cycle progression pathway by PLK1 inhibition and TRAIL apoptotic pathway, might have synergistic therapeutic efficacy. Although interesting clinical results suggest a possible role for TRAIL and PLK1 inhibition as monotherapy in cancer therapy, combined use of TRAIL receptor agonists and PLK1 inhibitors has not yet been investigated.

## RESULTS

### Effect of rhTRAIL and PLK1 inhibitor monotherapy in NSCLC cells

**Figure 1:**
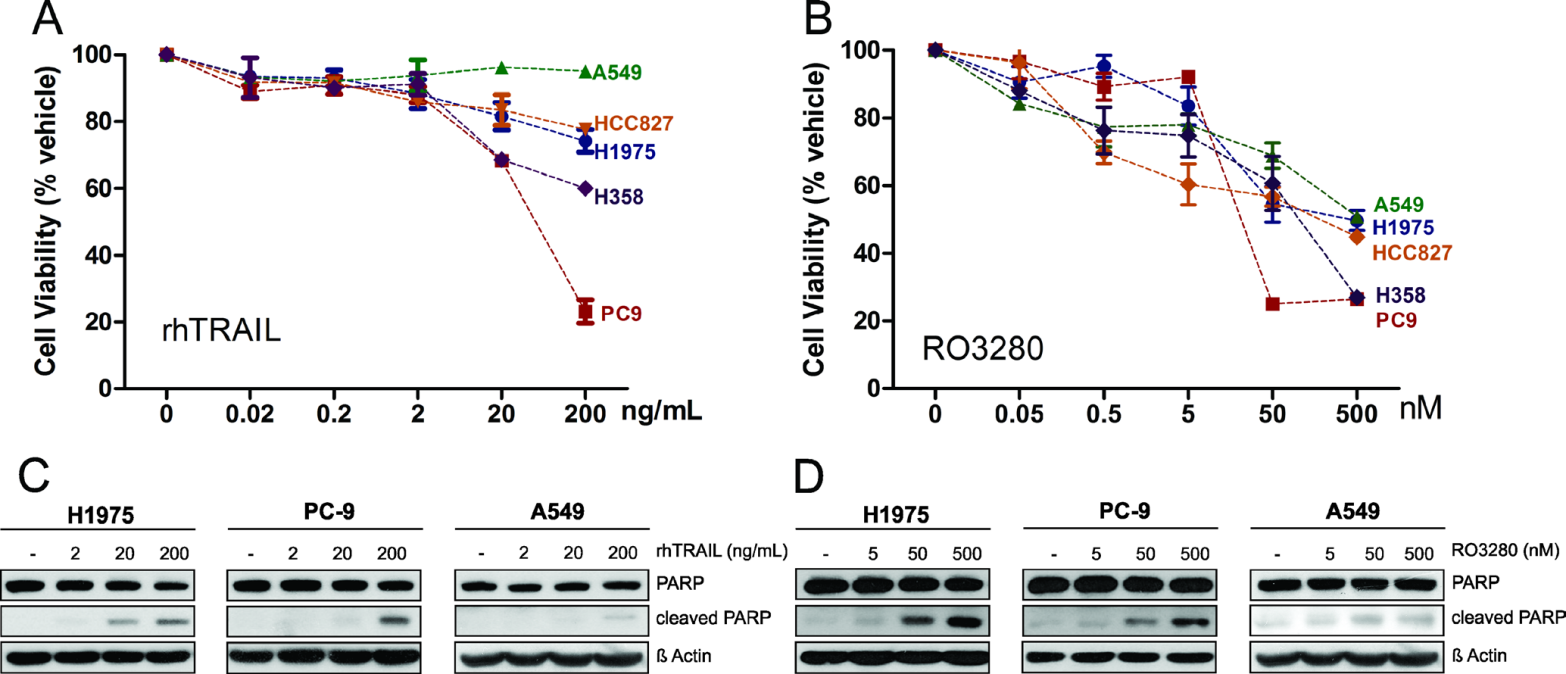
Cytotoxicity of rhTRAIL or PLK1 inhibitor RO3280 monotherapy in NSCLC cells H1975, PC9, HCC827, A549 and H358 cells were treated with increasing concentrations of rhTRAIL (0.02, 0.2, 2, 20, 200 ng/ml) (**A**) or PLK1 inhibitor RO3280 (0.05, 0.5, 5, 50, 500 nM) (**B**). Cell viability was measured using MTS assay after 72 hours incubation (*n =* 4, mean ± SD) (A, B). H1975, PC9 and A549 cells treated with 5, 50, 500 nM RO3280 or 2, 20, 200 ng/ml rhTRAIL for 24 hours were prepared for western blot analysis to determine PARP cleavage levels (**C**, **D**).

We first examined the effects of rhTRAIL and the PLK1 inhibitor RO3280 as single agents in various NSCLC cell lines with different genetic backgrounds (H1975, PC9, HCC827, A549 and H358; see Table 1). The cells were treated with rhTRAIL in a concentration range of 0.02–200 ng/ml or with RO3280 (0.05–500 nM) for 72 hours. The cell viability was measured (Figure 1A, 1B) and IC50 values were calculated from the dose curve using the Compusyn software (Supplementary Figure 1). As summarized in Table 1, PC9 cells were the most sensitive to rhTRAIL, while the other investigated cell lines exhibited intermediate-sensitivity or resistance to TRAIL with IC50 values above 200 ng/ml. The majority of the tested cell lines exhibited high sensitivity or intermediate-sensitivity to RO3280 with varying IC50 values. However, the A549 cell line demonstrated resistance to RO3280 with an IC50 concentration above 500 nM.

**Table 1:** Effect of rhTRAIL and PLK1 inhibitor in different NSCLC cell lines

Cell lines	Oncogene mutation status	RO3280 IC50 (nM)	rhTRAIL IC50 (ng/mL)
**PC9**	EGFR mutation (delE746-A750)	50	30.74
**H358**	KRAS mutation (G12C)	58	>200
**HCC827**	EGFR mutation (delE746-A750)	69	>200
**H1975**	EGFR double mutation (p. L858R/T790M)	224	>200
**A549**	KRAS mutation (G12S)	>500	>200

We further investigated three of these cell lines with representative genotypes: PC9 cells containing a single epidermal growth factor receptor (EGFR) mutation, H1975 cells containing a double EGFR mutation and A549 cells harboring a K-Ras mutation. The apoptotic effect of rhTRAIL (2–200 ng/ml) and RO3280 (5–500 ng/ml) as single therapy was tested in the three NSCLC cell lines by examining poly (ADP-ribose) polymerase (PARP) cleavage. As shown in Figure 1C, rhTRAIL induced PARP cleavage in a dose dependent manner in TRAIL-sensitive PC9 cells and TRAIL-resistant H1975 cells. Single treatment with rhTRAIL resulted in low PARP activity in A549 cells, the most resistant of the tested cell lines. Treatment with RO3280 induced PARP cleavage in H1975 and PC9 cells in a dose-dependent manner, but to a lesser extent in A549 cells (Figure 1D).

### RO3280 in combination with rhTRAIL synergistically reduces cell viability in NSCLC cells

**Figure 2:**
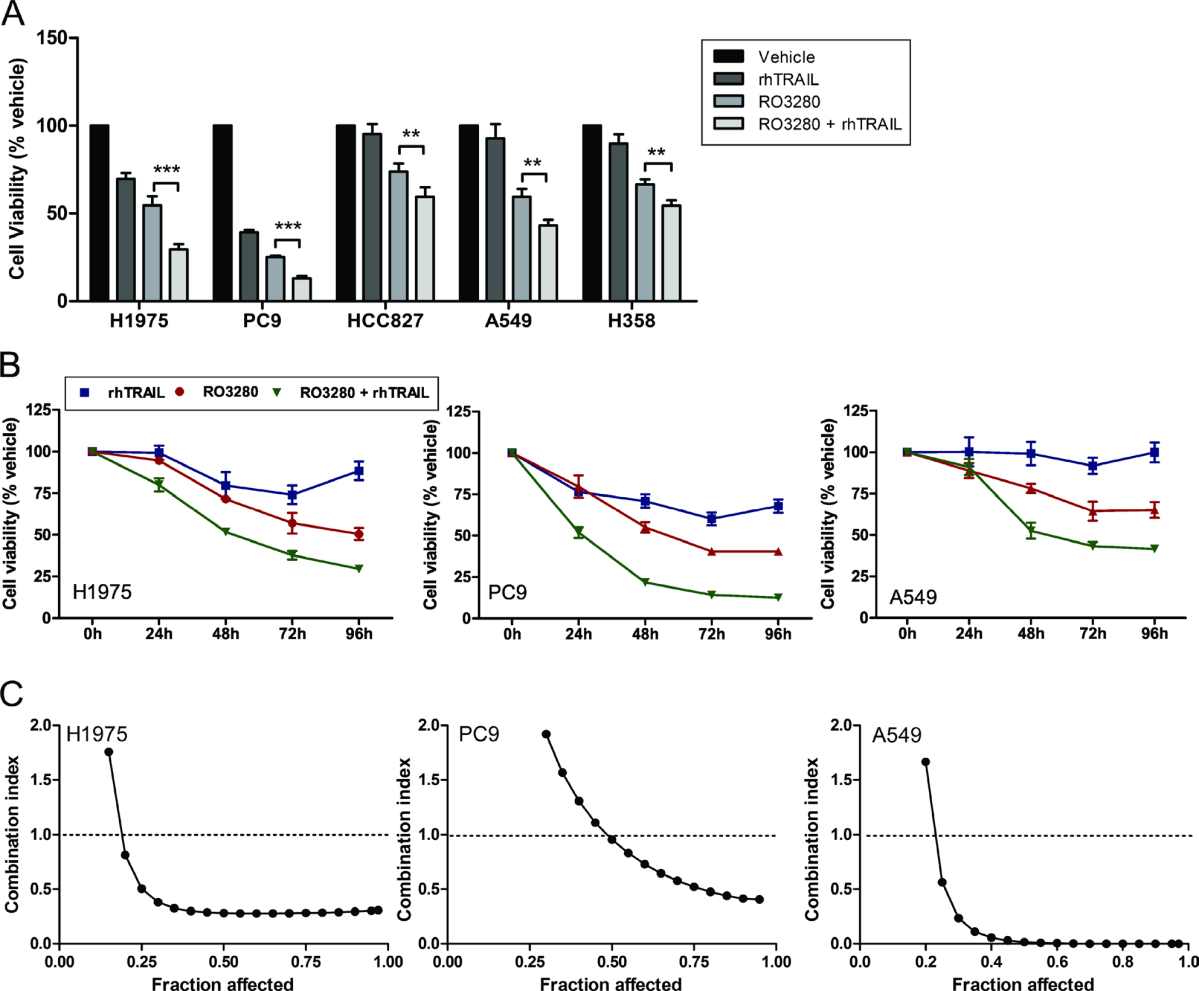
Synergistic effect of RO3280 and rhTRAIL combined treatment in NSCLC cells Cells were cultured simultaneously with 50 nM RO3280 and 20 ng/ml rhTRAIL (**A**, **B**) and an increased concentration of RO3280 (nM) and rhTRAIL (ng/ml): 1) 0:0; 2) 0.05:0.02; 3) 0.5:0.2; 4) 5:2; 5) 50:20; 6) 500:200; 7) 5000:2000 (**C**). Cell viability was analyzed by MTS assays after 72 hrs incubation (A) or at indicated time points (*n =* 4, mean ± SD) (B). The combination index/fraction affected curve was calculated with the Compusyn program (C).

Next, we examined whether we could increase the sensitivity of NSCLC cells to TRAIL-induced anti-tumor activity by testing a combination of rhTRAIL (20 ng/mL) and RO3280 (50 nM) in all five NSCLC cell lines. Statistical tests revealed in all cell lines a significant reduction of cell viability when cells were treated with the drug combination compared to single agent treatments (Figure 2A).

We further investigated this drug combination in a time-course experiment. H1975, PC9 and A549 cells were simultaneously treated with RO3280 (50 nM) and rhTRAIL (20 ng/ml) for 24, 48, 72, and 96 hours respectively. The result demonstrates that the combined treatment reduces cell viability in a time-dependent manner in the three cell lines (Figure 2B).

To ascertain the additive or synergistic nature of this drug combination, we calculated the combination index (CI) [32]. RO3280 (0.05–500 nM) was combined with rhTRAIL (0.02–200 ng/ml) at a constant ratio in H1975, PC9 and A549 cells. Cell viability was assessed after 72 hours and the CI and fraction affected curve was calculated using the Compusyn software. Synergistic effects were observed at IC50/ED50 in all cells, with strong synergism (CI = 0.1–0.3) in H1975 and very strong synergism (CI < 0.1) in A549 cells respectively (Figure 2C).

### RO3280 enhances TRAIL-mediated apoptosis in NSCLC

**Figure 3:**
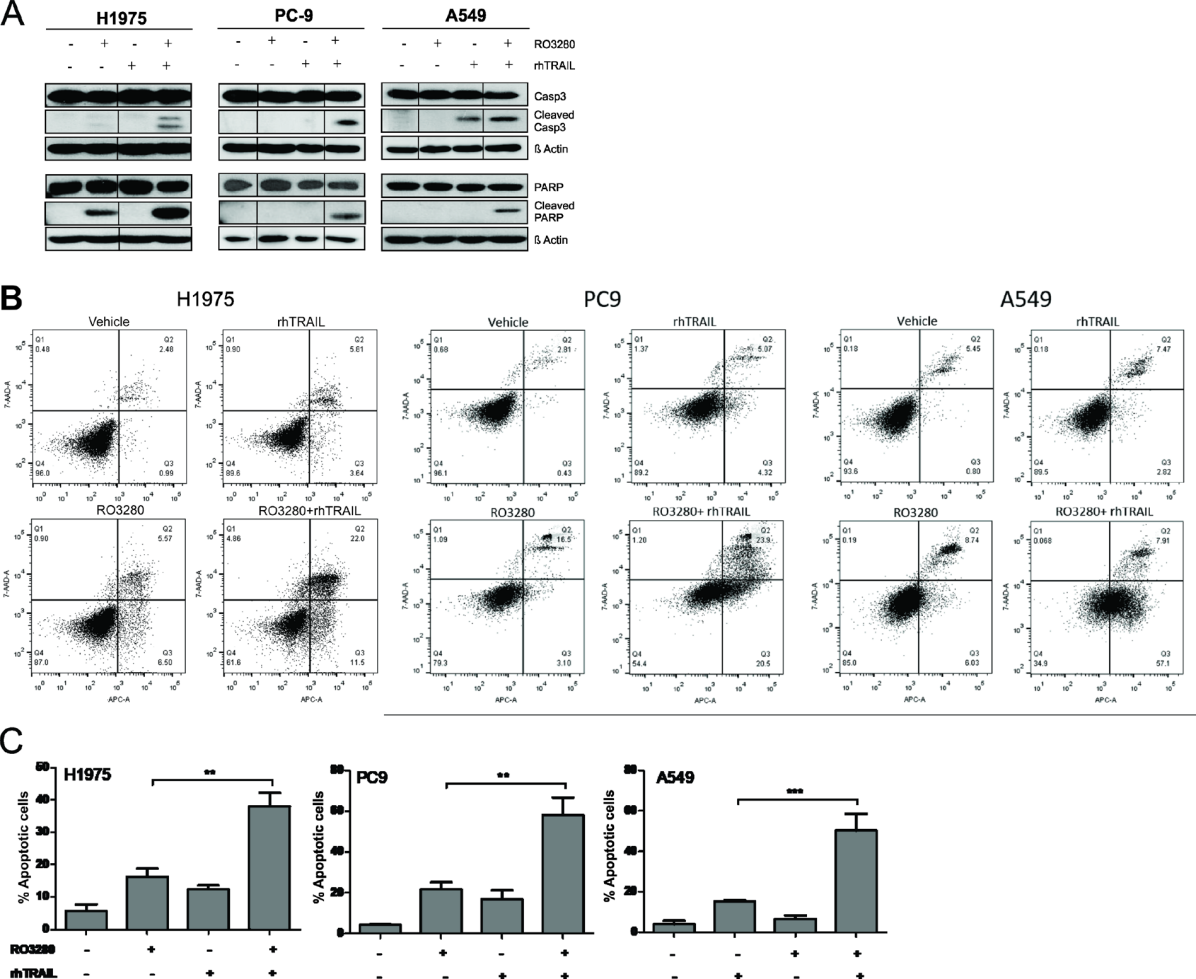
PLK1 inhibition by RO3280 increase TRAIL-induced apoptosis in NSCLC cells H1975, PC9, and A549 cells were treated with a combination of RO3280 (50 nM) and TRAIL (20 ng/ml) for 24 h. PARP/caspase-3 activity was analyzed by western blot. For each cell lines, lysates were run in the same gels (**A**). Representative image of apoptotic activity measured by flow cytometry (Q1: necrotic cells; Q2: late apoptotic cells; Q3: early apoptotic cells; Q4: viable cells;) (**B**). Indicated percentage of apoptotic cells represent early as well as late apoptotic cells and are derived from 3 independent experiments (± SEM) (**C**).

Apoptotic activity was assessed by examining caspase-3 and PARP cleavage by western blot analysis. As shown in Figure 3A, caspase-3 activity was increased in H1975, PC9 and A549 cells treated with the combination of RO3280 (50 nM) and rhTRAIL (20 ng/ml) compared to control and single agent exposure. A similar result was also observed for PARP, where the combination treatment increased PARP cleavage in all tested cells (Figure 3A).

In addition, we confirmed apoptotic activity by examining the number of annexin-V positive cells via flow cytometric analysis (Figure 3B). Further statistical analysis from at least three independent experiments shows that cells treated with rhTRAIL alone demonstrated an apoptotic rate of approximately 7 to 17% while exposure to RO3280 induced an apoptotic rate of approximately 15 to 22% in the investigated cell lines (Figure 3C). Significantly higher level of apoptotic activity of approximately 38 to 58% was observed in all cells treated with the drug combination.

### PLK1 inhibition sensitizes cells to TRAIL-mediated apoptosis by blocking the cell cycle

**Figure 4:**
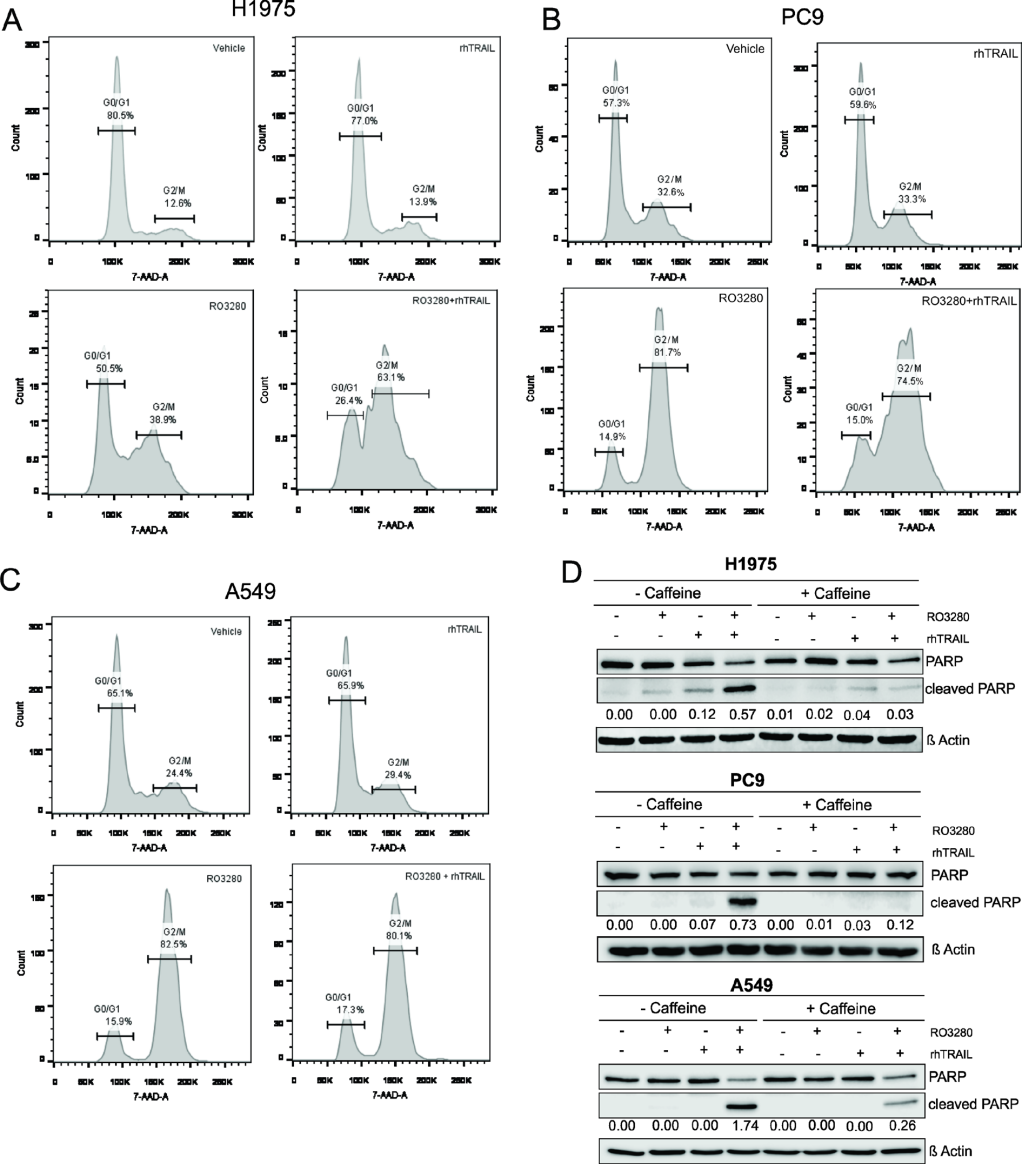
RO3280-induced G2/M cell cycle arrest sensitizes NSCLC cells to TRAIL-induced apoptosis H1975, PC9 and A549 cells were treated with 50 nM RO3280 and 20 ng/ml rhTRAIL for 24 hrs. Cells were stained with 7-AAD and the cell cycle was analyzed by flow cytometry (**A**–**C**). Cells were treated with or without caffeine (300 µg/ml for H1975 and A549, 600 µg/ml for PC9 in serum free medium) for 12–18 hrs, followed by RO3280 and rhTRAIL treatment for 24 hrs. Cell lysates were analyzed by western blot with indicated antibodies. PARP cleavage normalized by β-Actin were quantified by Image studio (**D**).

The effect of the drug combination on the cell cycle was assessed by flow cytometry in NSCLC cell lines. As shown in Figure 4A–4C, rhTRAIL did not induce cell cycle arrest in H1975, PC9 and A549 cells. However, PLK1 inhibitor treatment resulted in an increase in the distribution of cells at the G2/M phase in all three cell lines, indicating cell cycle arrest at G2/M. PLK1-induced G2/M cell cycle arrest was not further affected when the cells were additionally treated with rhTRAIL in PC9 and A549 cells. Interestingly, in H1975 cells, the number of cells in the G2/M phase markedly increased in the combined treatment condition (Figure 4A).

To further investigate whether cell cycle inhibition is the mechanism involved in the synergism observed between PLK1 and rhTRAIL, caffeine was used to prevent G2/M cell cycle arrest. H1975, PC9, and A549 cells were pre-treated with caffeine (300 µ/ml in H1975 and A549 and 600 µ/ml in PC9) for 12–18 hrs followed by RO3280 and rhTRAIL treatment for 24 hrs. The results indicate that caffeine addition induced a G0/G1 arrest (Supplementary Figure 2). Apoptotic activity was further examined by PARP cleavage analysis in caffeine-treated and untreated cells. The result shows that PARP cleavage was markedly reduced in all caffeine-treated cells followed by RO3280 and rhTRAIL treatment (Figure 4D). Overall, these results suggest that cell cycle blocking in the G2/M phase potentially contributes to the sensitization of cells to TRAIL-induced apoptosis.

### Effect of RO3280 in combination with rhTRAIL on the TRAIL apoptotic pathway

To further decipher the molecular mechanism by which RO3280 and rhTRAIL induce apoptosis, we examined the effects of the drugs on the expression of the TRAIL receptors TRAIL-R1 (Death receptor 4/DR4) and TRAIL-R2 (Death receptor/DR5), as well as on the cellular FLICE-like inhibitory protein (FLIP) protein that is known to control the classical death receptor-mediated extrinsic apoptosis pathway. Our results indicate that RO3280 single treatment or RO3280/rhTRAIL combination treatment slightly increases DR5 protein levels in all the tested cell lines (Supplementary Figure 3). Differential effects were observed on DR4 and FLIP in the treated cells lines. DR4 levels were increased in H1975 cells treated with RO3280 or RO3280/rhTRAIL but not in other tested cells lines. Similar treatment resulted in the reduction of FLIP expression levels in PC9 and A549, but not in H1975 cells.

**Figure 5:**
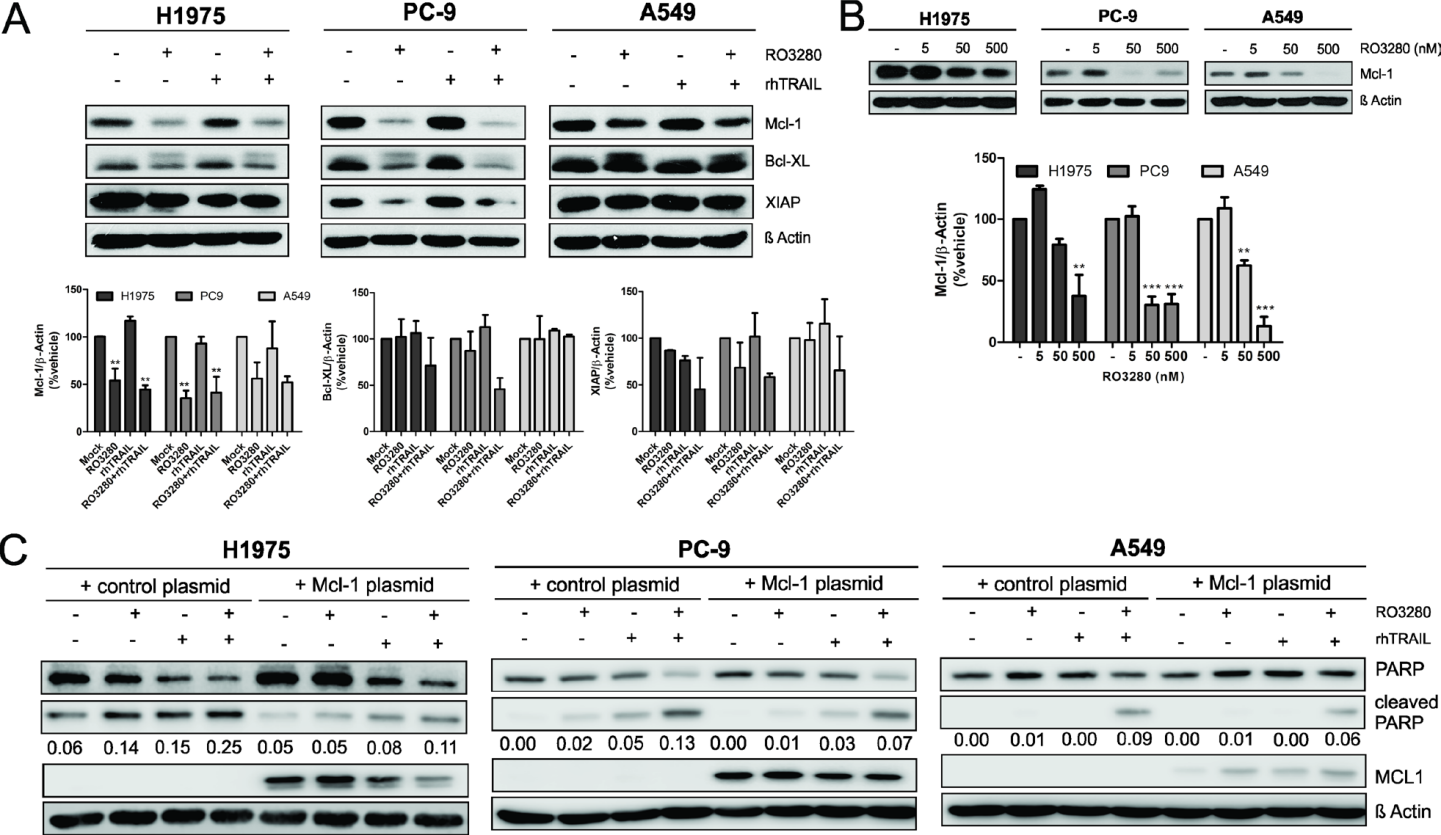
Mcl-1 downregulation by RO3280 increase TRAIL apoptotic activity in NSCLC cells Cells were cultured with 50 nM RO3280 and 20 ng/ml rhTRAIL (**A**) or 5, 50, 500 nM RO3280 for 24 h (**B**) The cell lysates were analyzed by western blot with indicated antibodies and immunoblot bands values are shown in histograms (*n =* 3, ± SEM) (A, B). Cells were transfected with an Mcl-1 encoding plasmid or a control plasmid 24 hrs prior to treatment (**C**). The cells were further treated with RO3280 (50 nM) and rhTRAIL (20 ng/ml). After 24 hrs, cell lysates were analyzed by western blot. PARP cleavage normalized by β-Actin (C).

We then examined the effect of RO3280 and TRAIL on the expression of anti-apoptotic proteins such as x-linked inhibitor of apoptosis protein (XIAP) and the Bcl-2 protein family members Bcl-XL and Mcl-1. XIAP plays a crucial role in the TRAIL-induced apoptotic pathway as it counteracts apoptosis by directly inhibiting caspase-3 and caspase-9 [33]. Downregulation of Mcl-1 was significantly observed in all three cell lines treated with RO3280 without an additional effect when combined with rhTRAIL (Figure 5A). Only a slight downregulation of the Bcl-XL and XIAP protein was observed in PC9 cells treated with RO3280 alone or in combination with rhTRAIL (Figure 5A). Our result further demonstrates that RO3280 inhibits Mcl-1 protein expression in a dose-dependent manner (Figure 5B), but not Bcl-XL and XIAP protein expression (data not shown).

We then sought to define whether Mcl-1 downregulation by PLK1 inhibition also plays a role in the synergistic effect of combined RO3280 and rhTRAIL exposure. H1975, PC9 and A549 cells were transfected with an Mcl-1 encoding plasmid or a control plasmid. After 24 hrs of single or combined treatment, cells were harvested and assessed for apoptotic activity by measuring PARP cleavage. The results, as shown in Figure 5C, indicate that PARP cleavage was reduced in all Mcl-1 overexpressing cells treated with combined treatment.

### Effect of RO3280 in combination with rhTRAIL on the survival pathway of NSCLC

**Figure 6:**
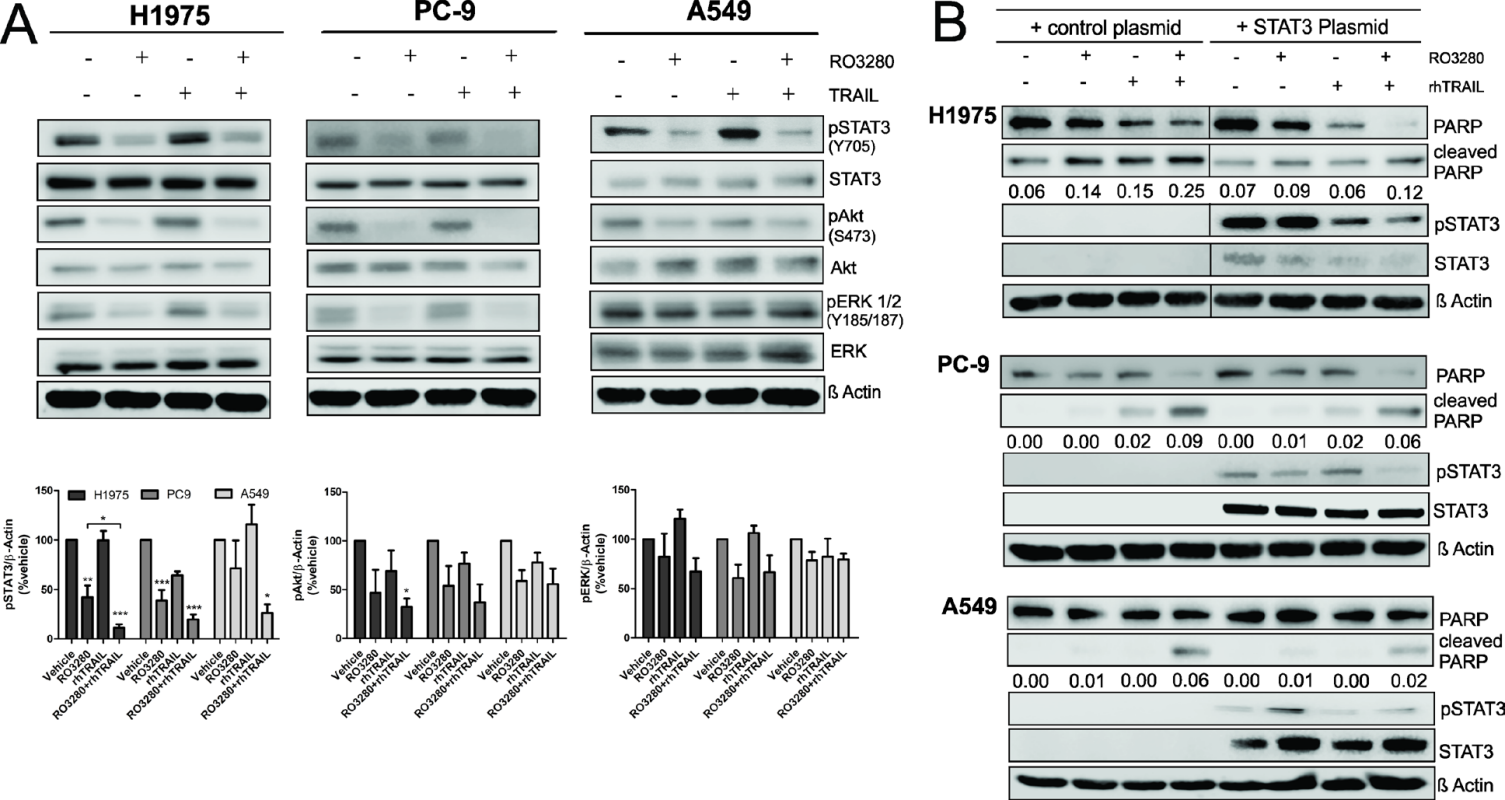
The influence of RO3280/rhTRAIL combined treatment on the signaling pathway in NSCLC cells H1975, PC9, and A549 cells were treated with a combination of RO3280 (50 nM) and rhTRAIL (20 ng/ml) for 24 hrs and cell lysates were analyzed by western blot with indicated antibodies (**A**). Quantification of immunoblot analysis blots are shown in histogram (*n =* 3, ± SEM) (A). Cells were transfected with STAT3 plasmid or a control plasmid 24 hrs prior to drugs treatment and were further treated with RO3280 (50 nM) and rhTRAIL (20 ng/ml) (**B**). After 24 hrs, cell lysates were analyzed by western blot with indicated antibodies (B). PARP cleavage normalized by β-Actin were quantified by Image studio. Cell lysates for each cell line were run in the same gels (B).

As the combination of RO3280 and rhTRAIL can synergistically reduce cell viability and induce apoptosis in NSCLC cells, we sought to further investigate additional relevant signaling pathways, such as the mitogen-activated protein kinases/extracellular signal-regulated kinases (MAPK/ERK), the Akt and, the janus kinase/signal transducers and activators of transcription (JAK/STAT) pathways, which play an essential role in the survival of NSCLC [34]. As shown in Figure 6A, treatment with RO3280 resulted in the downregulation of phosphorylated STAT3. Surprisingly, statistical analysis further shows that phosphorylated STAT3 downregulation in cells that were treated with the combination of RO3280 and rhTRAIL was significantly higher compared to single treatment. There was also a trend of Akt and ERK dephosphorylation in H1975 and PC9 treated with RO3280. However, the data were not statistically significant, except Akt activity downregulation in H1975 treated with RO3280 and rhTRAIL.

To further confirm that the observed downregulation of STAT3 activity results from the synergistic effect of RO3280 and rhTRAIL, we transiently overexpressed STAT3 in H1975, PC9 and A549 cells followed by treatment with RO3280, rhTRAIL or their combination. As shown in Figure 6B, overexpression of STAT3 in RO3280/rhTRAIL treated cells reduced the amount of cleaved PARP, compared to cells transfected with control plasmid. In STAT3 overexpressing cells, Mcl-1 was not detected (data not shown).

### PLK1 inhibition by volasertib sensitizes cells to TRAIL-mediated apoptosis *in vivo*

In the final part of the study we examined the effect of PLK1 inhibition on TRAIL-induced apoptosis *in vivo*. Volasertib, a PLK1 inhibitor that has been widely used in clinical trials was chosen to evaluate the PLK1 inhibition combined with rhTRAIL in a murine model of NSCLC. First, the combination of volasertib and rhTRAIL was verified in H1975, PC9 and A549 cells *in vitro*, and the results demonstrate the combination of volasertib and rhTRAIL reduces cell viability and increase apoptosis in all the tested cell lines (Supplementary Figure 4).

**Figure 7:**
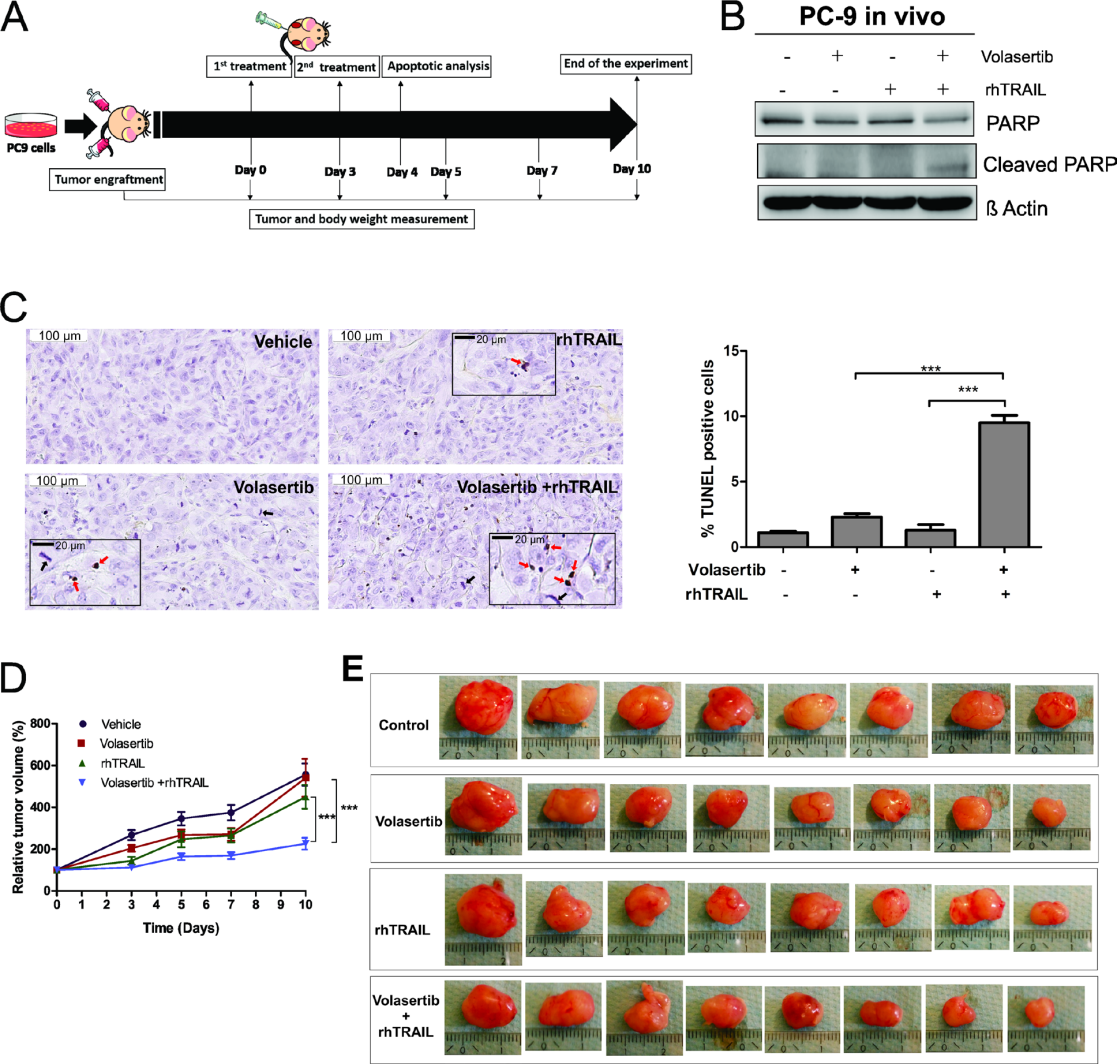
PLK1 inhibition increase TRAIL-induced apoptosis on xenograft model Nude mice carrying PC9 tumors on left and right flanks (10 tumors per group, 17 tumors for control group) receive vehicle (4% dimethyl sulfoxide (DMSO) in corn oil), volasertib (20 mg/kg) or rhTRAIL (10 mg/kg) on day 0 and 3 through intraperitoneal injection (**A**). On day 4, representative mice from each group were sacrificed and tumors were excised. Two representative tumors from each group were analyzed for apoptotic activity by western blot using indicated antibody (**B**) and TUNEL assay (**C**). Representative field (Scale bar: 100 µm) with insert magnification (Scale bar: 20 µm) (Red arrows: TUNEL positive cells; Black arrows: mitotic spindle) (C). The percentage of TUNEL positive cells (% total cells) were calculated from 10 random fields (*n =* 10, mean ± SEM) (C). Also see Supplementary Figure 5 for mitotic spindle calculation. Relative tumor volume was measured (mean ± SEM) for 10 days. The relative tumor volume in the treatment groups (*n =* 8 tumors) versus control group (*n =* 15 tumors) was compared using linear regression, with best fit values (**D**). Visualization of excised tumors from all treatment groups (**E**).

We generated xenograft tumors by injecting subcutaneously PC9 cells into nude mice. When the tumors reached approximately 80–100 mm^3^, the mice were randomly assigned into four treatment groups (Vehicle control, volasertib, rhTRAIL and volasertib+ rhTRAIL groups). The mice were subsequently treated twice with the drugs (see Material and Methods and Figure 7A). The apoptotic activity was examined 24 hrs after the second treatment in two representative tumors from each group. We observed increased PARP cleavage in tumors that were treated with volasertib and rhTRAIL combined, but not in control or single treatments (Figure 7B). This result was further confirmed by the terminal deoxynucleotidyl transferase (TdT) dUTP nick-end labeling (TUNEL) assay, demonstrating a significant increase of TUNEL positive cells in the combination group (Figure 7C). From TUNEL assay tumor slides in Figure 7C, we also observed increased amounts of mitotic spindles in tumors that were treated with volasertib (4.8 ± 0.59%) or in combination with rhTRAIL (5.6 ± 0.64%) compared to rhTRAIL (1.5 ± 0.27%) and control (1.2 ± 0.33%) (Supplementary Figure 5).

Tumor volume was monitored over time, and the relative tumor volume was calculated as the difference between the tumor volume after treatment compared to tumor volume at day 0 (start of the treatment day). Volasertib (Slope: 40 ± 6.13%) and rhTRAIL (Slope: 34 ± 4.66%) single treatment did not significantly decrease relative tumor volume compared to vehicle control (Slope: 43 ± 4.43%). However, combined treatment with volasertib and rhTRAIL (Slope: 12.8 ± 2.16%) significantly decreased the relative tumor volume compared to control and single treatments (Figure 7D and 7E). In addition, the weight of nude mice did not exhibit a significant change during experiments (Supplementary Figure 6), suggesting that single or combination treatment with volasertib and/or rhTRAIL, do not cause toxicity at used doses in mice.

## DISCUSSION

Targeting cell cycle progression is considered as one of the possible mechanism to sensitize tumor cells to TRAIL-induced apoptosis [17]. However, this approach has not been tested in NSCLC cells. PLK1 is the best characterized member of the polo-like kinase family that is considered as an emerging anti-mitotic target for NSCLC [35]. While PLK1-directed monotherapy has demonstrated manageable side effects but limited clinical efficacy, combination therapy is considered as a potential strategy to improve drug efficacy. In the current work we show for the first time, that combining a PLK1 inhibitor with rhTRAIL significantly promotes apoptosis *in vitro*. We subsequently demonstrated that the combined drug treatment significantly increases apoptosis and suppresses tumor growth in an *in vivo* xenograft model.

Inhibition of PLK1 kinase activity by PLK1 inhibitors such as volasertib, BI 2536, and GSK461364 has been shown to induce cell cycle arrest at the G2/M phase in glioblastoma cells, osteosarcoma and NSCLC cells [30, 31, 36–38]. In our study, we showed that the fraction of NSCLC cells in the G2/M phase increased upon inhibition of PLK1 indicating a cell cycle arrest in this phase. Additionally, using a xenograft model of NSCLC we demonstrated that PLK1 inhibition increases the occurrence of mitotic spindles, which further deciphers that PLK1 inhibition induces cell cycle arrest possibly at the mitotic level. Indeed, our results are in agreement with investigations by Zhang *et al.* which revealed that blocking PLK1 kinase activity induces G2/M arrest and mitotic phase arrest in bladder carcinoma cell lines [39]. We further demonstrated that inducing G0/G1 arrest with caffeine in NSCLC cells, markedly suppresses the synergistic effect of a PLK1 inhibitor combined with a TRAIL agonist. Taken together, our results provide support for the conceptual premise that G2/M cell cycle arrest, possibly at the mitotic phase, is beneficial for the sensitization to TRAIL-induced apoptosis in lung cancer cells.

The balance between apoptosis and cell cycle regulators may influence the destiny of cell homeostasis, with improper regulation, both mechanisms could contribute to pathological conditions including cancer. It is likely that these two mechanisms are directly or indirectly linked, as several genes involved in cell cycle regulation such as p53, Myc and pRb are also involved in apoptosis [40, 41]. Cell cycle arrest mediated by p53 has been previously described as a mechanism by which cytotoxic drugs sensitize tumor cells to TRAIL-induced apoptosis [17]. Hence future studies may focus on p53 to reveal the full mechanisms of how cell cycle arrested by PLK1 could increases TRAIL-induced apoptosis.

TRAIL triggers the apoptotic pathway by binding to its trans-membrane receptors DR4 and DR5, inducing apoptotic signals via DISC and caspase-8 activation (Type I), or via the mitochondrial pathway (Type II) if caspase-8 activation is not sufficient to induce apoptosis. We observed a slight increase in DR5 expression following PLK1 inhibition, however, it remains to be tested whether DR5 upregulation is involved in the sensitization of TRAIL-induced apoptosis. Another mechanism of PLK1-TRAIL synergy may be the inhibition of anti-apoptotic molecules in the mitochondria. The mitochondrial pathway is controlled by the pro-apoptotic Bcl-2 family members Bax and Bak, but also by anti-apoptotic Bcl-2 family members such as Mcl-1 and Bcl-XL [14, 15]. In response to PLK1 inhibition, our result demonstrate downregulation of Mcl-1, thereby increasing the sensitivity of NSCLC cells to TRAIL-induced apoptosis.

Another interesting finding is that phosphorylation of STAT3 was significantly inhibited when the NSCLC cells were treated with RO3280 in combination with rhTRAIL. To date, there are not many documented studies investigating the relationship between PLK1 with the STAT3 pathway. One study in esophageal cancer revealed that STAT3 and PLK1 control each other’s transcription in a positive feedback loop [42]. The study also demonstrated that RNA interference mediated PLK1 RNA inactivation led to a decrease in STAT3 transcriptional activity in esophageal cancer cells [42]. However, in the present study we found that the level of phosphorylated STAT3 was influenced by PLK1 inhibition while the total amount of STAT3 protein was unaffected. Our finding thus warrants further investigation on the mechanism by which PLK1 mediates STAT3 activity. Bcl-XL and Mcl-1 transcription are reported to be induced by activated STAT3 according to Rahaman *et al.* [43]. In addition, several other studies have also reported that TRAIL-mediated apoptosis is strongly increased when the anti-apoptotic Bcl-2 family member Mcl-1 is downregulated via JAK2-STAT3 inhibition [44–46]. PLK1/STAT3 inhibition can possibly activate the Type II mitochondrial pathway via Mcl-1 downregulation. However, our findings are not in agreement with the literature as Mcl-1 upregulation was not observed in STAT3-overexpressed NSCLC cells.

We found that a combination of PLK1 inhibitor and rhTRAIL significantly reduces tumor growth. However, it is important to bear in mind that the combined treatment in the murine xenograft NSCLC tumor model was given in a short period, and at the minimal dosage. The optimum dose for complete tumor regression must be further investigated.

In conclusion, the present study reveals that TRAIL holds great promise for the treatment of NSCLC when used as a combinatorial strategy. Our corroborative *in vivo* results further support this. More importantly, no fatalities or toxicity were observed in the murine xenograft model, indicating the safety of the two drugs combined. The strong synergy of PLK1 inhibition and TRAIL activation may also represent a new form of synthetic lethality in NSCLC cells. Thus, we provide a rationale for the further exploration of the therapeutic potential and safety of TRAIL and PLK1 combination therapy in the treatment of NSCLC.

## MATERIALS AND METHODS

### Cell culture and reagents

The Human NSCLC cell lines NCI-H1975, HCC827 and H358 were obtained from the American Type Culture Collection (ATCC), while PC9 and A549 were obtained from Sigma. These cell lines were chosen based on their expression of mutated genes which are clinically relevant and represent the common heterogeneity of the disease. Cells identity was confirmed by STR analysis. All cells were cultured at 37° C in a humidified 5% CO_2_ incubator. H1975, PC9, HCC827 and H358 cells were grown in RPMI-1640 medium (Invitrogen) supplemented with 10% heat inactivated fetal bovine serum (FBS) (Perbio Science), 2 mM L-glutamine and 25 mM HEPES. A549 cells were grown in DMEM supplemented with 10% heat inactivated FBS, 2 mM L-glutamine and 25 mM HEPES. Stock Solution (10 mM) of RO3280 (Selleckchem) were prepared in dimethyl sulfoxide (DMSO) and stored at −80° C, further diluted in fresh medium prior to each experiment with the final concentration of DMSO less than 0.1%. Recombinant human TRAIL/Apo2 ligand was purchased from Peprotech and diluted in phosphate buffer saline (PBS).

### Cell viability assay

Cell viability was evaluated by the 3-(4,5-dimethylthiazol-2-yl)-5-(3-carboxymethoxyphenyl)-2-(4-sulfophenyl)-2H-tetrazolium) (MTS) assay (Cell Titer96 Aqueous One Solution Cell Proliferation Assay, Promega). Cells were seeded into each well of 96 well plates and allowed to attach for 24 hours prior to the addition of DMSO or drugs. After drug treatment, 20 µl MTS solution was added to each well followed by 2 hours incubation. Cell viability was determined by measuring the absorbance at 490 nm with a 96 well microplate reader (Scientific Multiskan MK3, Thermo). Four replicate wells were utilized for each analysis and at least three independent experiments were conducted.

### Annexin V staining

Cells were collected, washed with ice cold PBS and re-suspended in binding buffer (BD Biosciences). For apoptosis analysis the cells and supernatant were collected and were stained with APC fluorochrome-labeled 5 µl Annexin V (BD Biosciences) combined with 5 µl 7-Amino-Actinomycin (7-AAD) (BD Biosciences) to detect dead and damaged cells. The stained cells were analyzed by BD FACS Canto flow cytometry and were calculated using FlowJo^®^ software.

### Cell cycle analysis

Treated cells were trypsinized, washed with PBS and fixed in cold 70% ethanol. The single-cell suspensions were stained with 7-Amino-Actinomycin (7-AAD) (BD Biosciences) for subsequent analysis of the cell cycle by flow cytometry.

### Western blot analysis

Cultured cells were washed in ice-cold PBS and lysed in lysis solution containing Tris/HCL (pH 7.6) 20 mM, NaCl 150 mM (pH 6.85), EDTA 1 mM (pH 8), TRITON X 1%, Na-pyrophosphate 2.5 mM, sodium orthovanadate (Na3VO4) 1 mM, leupeptin 1 µg/ml, protease inhibitor cocktails 1% and phosphatase inhibitor cocktails 1% (Sigma). Protein concentration of the lysate was measured by the Bradford protein assay (Bio-Rad). Samples containing equal amounts of total protein were subjected to sodium dodecyl sulfate-polyacrylamide gel electrophoresis (SDS-PAGE). The resolved proteins were transferred from the gel to PVDF membranes and probed with indicated antibodies: STAT3, Akt, pSTAT3 (Tyr^705^, 3E2), ERK1/2, pERK (Tyr ^185/187^), caspase-3, PARP, DR5, DR4, XIAP, Bcl-XL and Mcl-1 from Cell signaling; pAkt (Ser^473^) from Invitrogen; FLIP from Abcam and β-Actin from Sigma. Detection of bound primary antibodies was performed with Horseradish peroxidase (HRP)-conjugated secondary antibodies (Anti-mouse or anti-rabbit IgG HRP linked, GE Healthcare) and visualized with the chemiluminescence detection kit (Western bright^™^ ECL, Advansta) with LI-COR Biosciences Odyssey^®^ Fc Imaging System and analyzed with Image Studio^™^.

### Plasmid transfection

The pTOPOMCL1 Plasmid (pcDNA3.1/V5HisTOPO backbone with CMV promotor) was a gift from Ulrich Maurer (Addgene plasmid # 21605). Cells were cultured in 96 wells plate 24 hours prior transfection. The plasmid (500 ng) was transfected using Lipofectamine^™^ 2000 (Invitrogen) in Opti-MEM (Gibco by life technologies), according to the manufacturer’s instructions. The transfected cells were cultured at 37° C for 24 hours and analyzed for analysis.

### Xenografts studies

All the procedures related to animal handling, care, and treatment were performed according to the guidelines approved by the University of Gent Animal Care and Use Ethical Committee. Athymic female nude mice (3–4 weeks) were purchase from Charles River Laboratories. PC9 Cells (2 × 10^6^) in 50% Matrigel were injected subcutaneously into right and left flanks of mice. Tumor-bearing mice were randomly assigned into four treatment groups (5 mice per group, 10 mice for control) and treatment initiated when xenografts reached volumes about 80–100 mm^3^. Tumors were measured using Vernier caliper and the volume was calculated using the formula: Volume = Length × Width × Width/2, where the width represents the shorter perpendicular dimension of the tumor. Volasertib was administered intraperitoneally (i.p.) at a dose of 20 mg/kg in corn oil (4% DMSO), while rhTRAIL was administered intraperitoneally at a dose of 10 m/kg in saline solution. For dual treatment groups, tumor-bearing mice received volasertib followed by rhTRAIL and control groups were given both vehicles

### TUNEL staining

Tumors were excised and fixed in 10% formalin. Tumor sections were analyzed for TUNEL activity using the “*In situ* Cell Death Detection Kit, POD” (Roche) according to the manufacturer’s instructions with hematoxylin counterstain. The percentage of apoptotic cells was calculated as the ratio of TUNEL positive nuclei to the total amount of nuclei. The percentage of mitotic spindles was calculated as the ratio of mitotic spindles to the total amount of nuclei.

### Statistical analysis

The results are representative of three independent experiments unless stated otherwise. Values were presented as the mean ± standard error of the mean (SEM). The unpaired two-tailed *t*-test was used to determine the significance of the differences between two groups. ANOVA followed by the Tukey test was done for studies with more than two groups. Relative tumor growth in the *in vivo* study was compared using linear regression, with best fit values. The difference between groups was considered statistically significant when *P* < 0.05 (^*^), *P* < 0.01 (^**^) and *P* < 0.001 (^***^).

### Analysis of drug combination effects

Analysis of drug combination was assessed by Compusyn software (ComboSyn, Inc.) as described [32]. The combination index (CI) was used to express synergism (CI < 1), additive effect (CI = 1) or antagonism (CI > 1) [47].

## SUPPLEMENTARY MATERIALS FIGURES



## Abbreviations

CI: Combination index
DISC: Death-inducing signaling complex
DR4/5: Death receptor 4/5
FLIP: cellular FLICE-like inhibitory protein
NSCLC: Non-small cell lung cancer
PARP: Poly (ADP-ribose) polymerase
PLK1: Polo like kinase 1
rhTRAIL: recombinant human TRAIL
STAT3: Signal transducer and activator transcription 3
TNF: Tumor necrosis factor
TRAIL: Tumor necrosis factor related apoptosis-inducing ligand
TRAIL-R1/2: TRAIL Receptor 1 or 2
XIAP: X-linked inhibitor of apoptosis protein
