# Supplementary material for: Targeting Polo-like kinase 1 and TRAIL enhances apoptosis in non-small cell lung cancer
Source: Oncotarget. 2018 Jun 19;9(47):28731–44. doi: 10.18632/oncotarget.25618 (PMC6033352; doi:10.18632/oncotarget.25618)
Supplement: Supplementary file 1 [file oncotarget-09-28731-s001.pdf]

Targeting Polo-like kinase 1 and TRAIL enhances apoptosis in non-small cell lung cancer

SUPPLEMENTARY MATERIALS

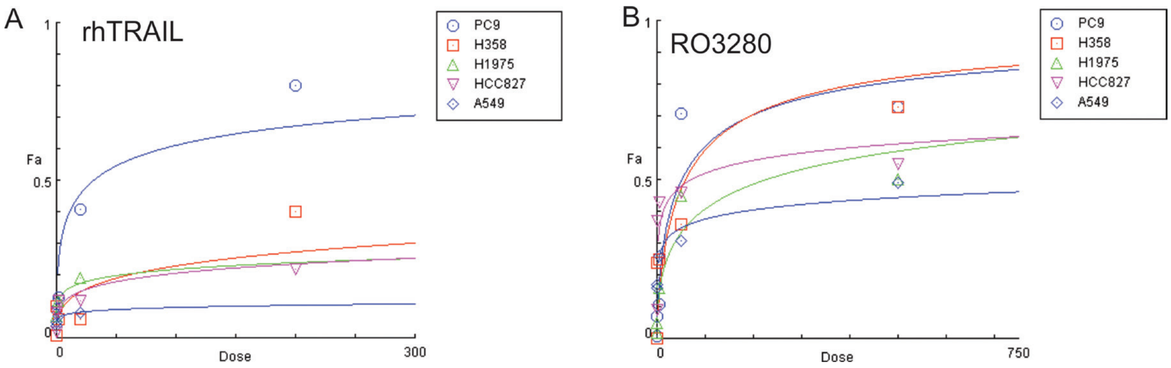

Supplementary Figure 1: RO3280 and rhTRAIL dose-response curve. H1975, PC9, HCC827, A549 and H358 cells were treated with increasing concentrations of rhTRAIL (0.02, 0.2, 2, 20, 200 ng/ml) (**A**) or PLK1 inhibitor RO3280 (0.05, 0.5, 5, 50, 500 nM) (**B**). Cell viability was measured using MTS assay after 72 hours incubation. Dose curves were calculated by Compusyn software.

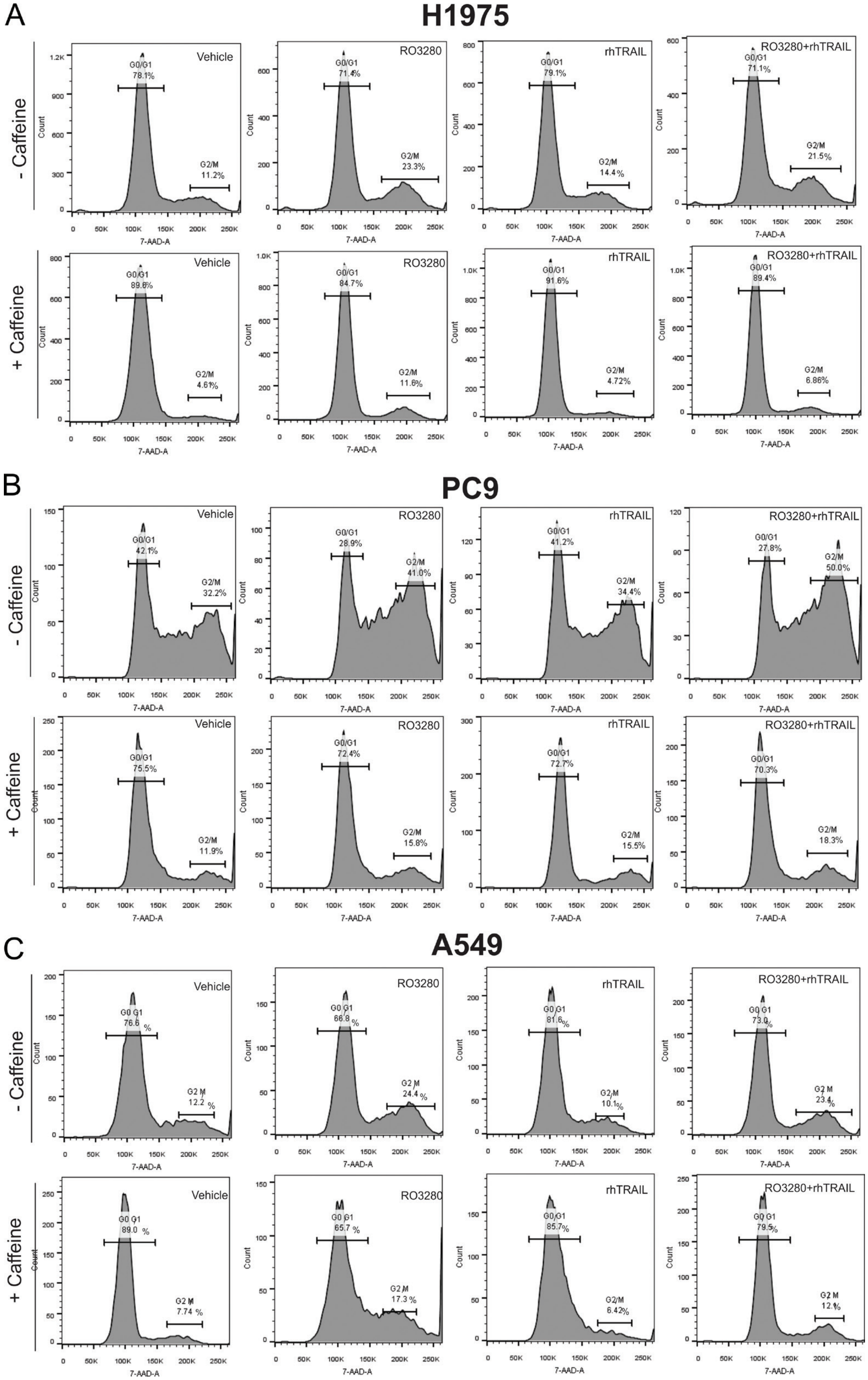

Supplementary Figure 2: Effect of caffeine in cell cycle progression on RO3280 and/or rhTRAIL treatment. Cells were treated with or without caffeine (H1975/A549: 300 µg/ml; PC9: 600 µg/ml) in serum free medium for 12–18 hrs, followed by RO3280 and rhTRAIL treatment for 24 hrs. Cells were stained with 7-AAD and the cell cycle was analyzed by flow cytometry (**A**, **B**, **C**).

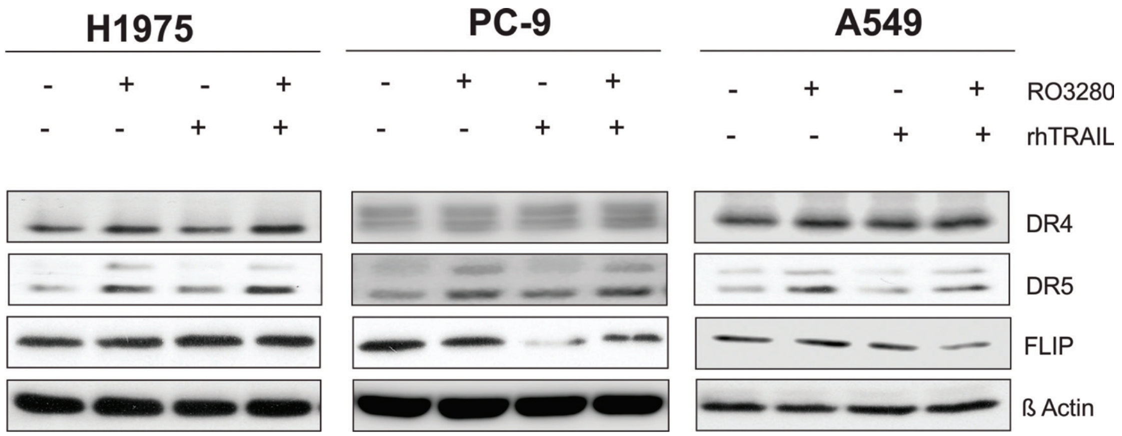

Supplementary Figure 3: Effect of RO3280 and rhTRAIL in Trail apoptosis pathway. Cells were cultured with 50 nM RO3280 and 20 ng/ml rhTRAIL for 24 hrs. Cell lysates were analyzed by western blot with indicated antibodies.

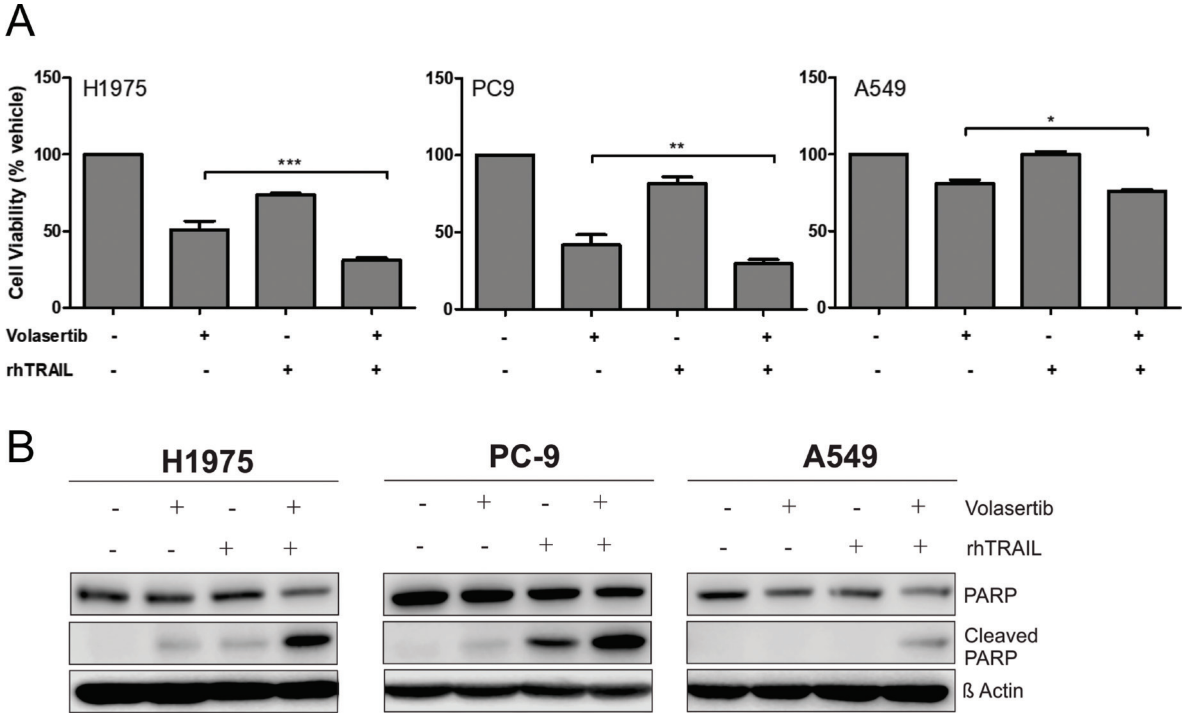

Supplementary Figure 4: Volasertib increase TRAIL-induced apoptosis in H1975, PC9 and A549 cells. H1975, PC9, and A549 cells were treated with 50 nM volasertib and 20 ng/mL rhTRAIL. Cell viability was analyzed after 72 hrs ($n = 4$, ± SEM) (**A**) and apoptotic activity was analyzed by western blot after 24 hrs with indicated antibodies (**B**).

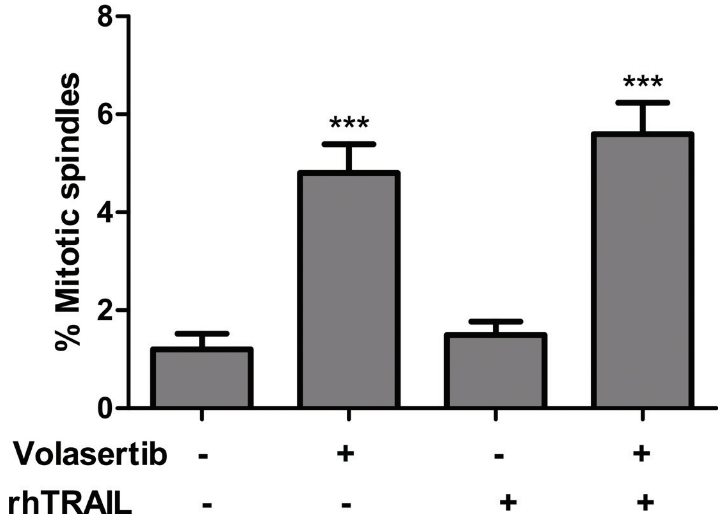

Supplementary Figure 5: PLK1 inhibition increases mitotic spindles in xenograft models. The percentage of mitotic spindles cells (% total cells) from Figure 7 were calculated from 10 random fields (*n* = 10, mean ± SEM).

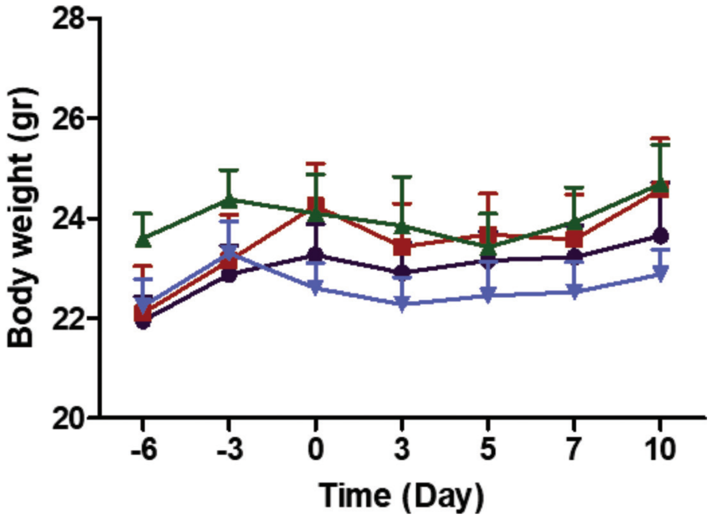

Supplementary Figure 6: Effect of a combination of volasertib and TRAIL on mice body weight. Body weight of mice from *in vivo* experiments were measured (mean ± SEM) for 10 days.
